# Non-coding RNAs and exosomal non-coding RNAs in lung cancer: insights into their functions

**DOI:** 10.3389/fcell.2024.1397788

**Published:** 2024-05-27

**Authors:** Xiaolong Lv, Lei Yang, Yunbo Xie, Mohammad Reza Momeni

**Affiliations:** ^1^ Department of Cardiothoracic Surgery, The People’s Hospital of Changshou, Chongqing, China; ^2^ Department of Cardiothoracic Surgery, The People’s Hospital of Tongliang District, Chongqing, China; ^3^ Department of Cardiothoracic Surgery, The First Affiliated Hospital of Chongqing Medical University, Chongqing, China; ^4^ School of Medicine, Tehran University of Medical Sciences, Tehran, Iran

**Keywords:** lung cancer, non-coding RNA, microRNA, long non-coding RNA, circular RNA

## Abstract

Lung cancer is the second most common form of cancer worldwide Research points to the pivotal role of non-coding RNAs (ncRNAs) in controlling and managing the pathology by controlling essential pathways. ncRNAs have all been identified as being either up- or downregulated among individuals suffering from lung cancer thus hinting that they may play a role in either promoting or suppressing the spread of the disease. Several ncRNAs could be effective non-invasive biomarkers to diagnose or even serve as effective treatment options for those with lung cancer, and several molecules have emerged as potential targets of interest. Given that ncRNAs are contained in exosomes and are implicated in the development and progression of the malady. Herein, we have summarized the role of ncRNAs in lung cancer. Moreover, we highlight the role of exosomal ncRNAs in lung cancer.

## Introduction

Lung cancer is one of the deadliest cancers and has a low 5-year survival rate, with only 22% of people affected surviving beyond that period. It is the most common form of cancer in both males and females (Siegel et al., 2018). The two varieties of lung cancer can be distinguished by the size of the cells that form them. The more prevalent type, non-small cell lung cancer, is seen in the majority of cases, around 85 percent. Small-cell lung cancer, though less common, still affects 15 percent of individuals (Inamura, 2017).

Lung cancer is a complicated disease, and it is brought about by a combination of genetic and epigenetic changes. This alteration permits pro-oncogenic and tumor suppressor genes, in addition to growth hormones, transcription factors, and other controlling molecules, to all factor into the rise of the cancer (Braicu et al., 2019). Recent studies on lung cancer have revealed the influential role of non-coding RNAs (ncRNAs). ncRNAs are pieces of RNA that do not form proteins but rather affect gene expression at both the transcriptional and post-transcriptional levels. These ncRNAs are separated into two classes: ones shorter than 200 base-pairs (bp) known as small non-coding RNAs and those longer than 200 bp referred to as long non-coding RNAs (Brosnan and Voinnet, 2009).

Exosomes are small extracellular sacs, measuring 40–160 nm across, which are created by invaginating the plasma membrane twice into numerous vesicles encased in a single intracellular container. The exosomes are eventually discharged by releasing these internal vesicles (ILVs) (Kalluri and LeBleu, 2020). Exosomes have been demonstrated to be produced and secreted from numerous types of cells, such as carcinoma cells (Wolfers et al., 2001), stromal cells (Fang et al., 2018), immune cells (Pitt et al., 2016), stem cells (Riazifar et al., 2019), and neurons (Fauré et al., 2006), etc. Research data aggregates to suggest that exosomes can act as messengers, ferrying intracellular material such as proteins, metabolites, amino acids, and DNA and RNA between cells (Kalluri and LeBleu, 2020). Previous investigations proposed that mRNA and miRNA were put into exosomes specifically to influence gene expression and oversee the activities of cells that received them (Valadi et al., 2007). Herein, we have summarized the role of ncRNAs in lung cancer. Moreover, we highlight the role of exosomal ncRNAs in lung cancer.

## MicroRNAs and lung cancer

**FIGURE 1:**
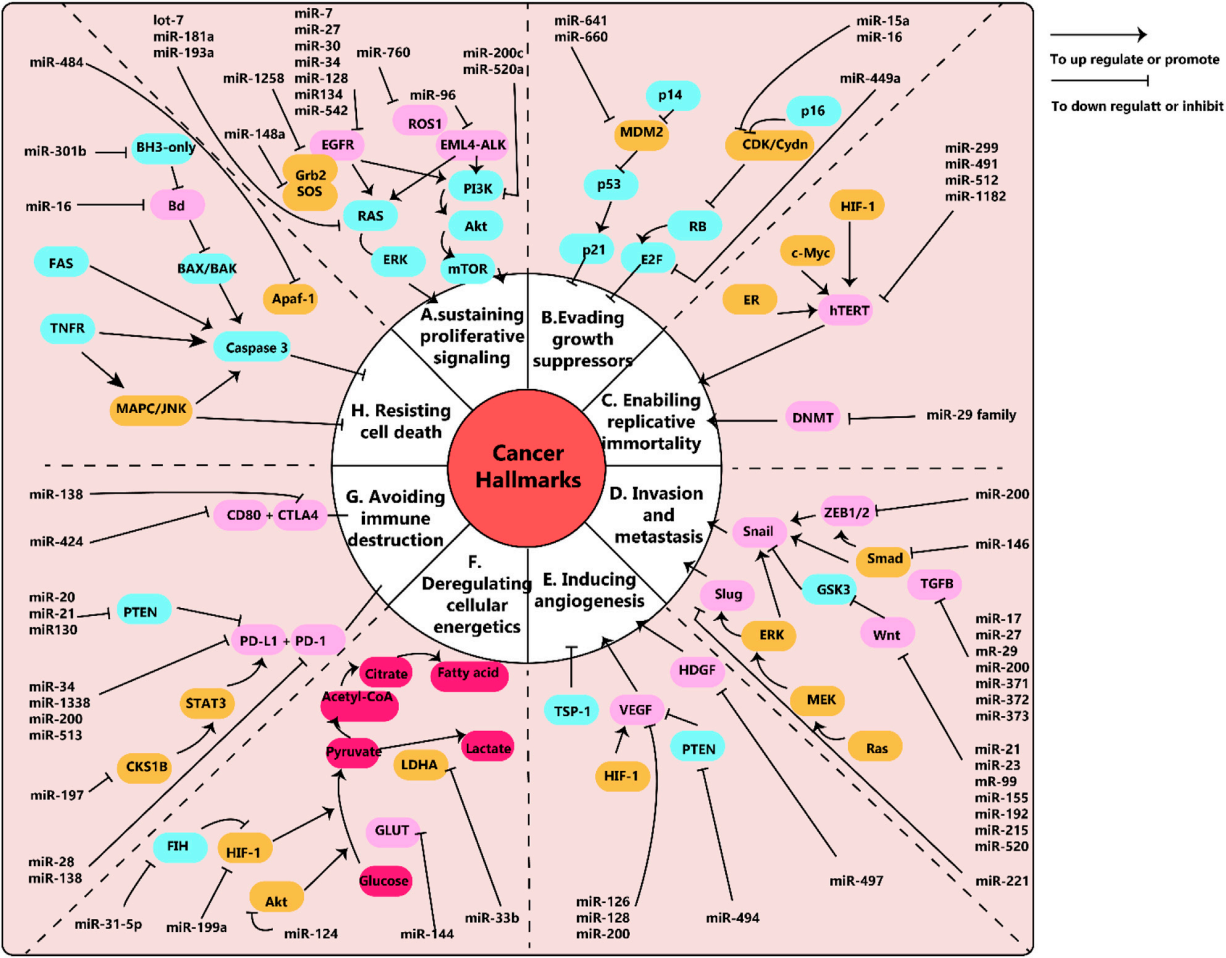
**(A)** schema of microRNAs and their pathways network in cancer. Epidermal growth factor (EGF) and its receptors, EGFR, work together to stimulate cell growth in lung cancer through the downstream RAS/ERK and PI3K/Akt/mTOR pathways. Similarly, EML4-ALK and ROS1 also trigger cancer cell growth through these pathways. Relevant miRNAs are depicted in the diagram. **(B)** Disruptions in tumor suppressors, RB and p53, enable cancer cells to avoid inhibition of growth. **(C)** The human telomerase reverse transcriptase (hTERT) is linked to the immortality of cancer cells. MiR-299, miR-491, miR-512, and miR-1182 have been found to target hTERT, but these findings have been studied in various cancer cells other than lung cancer. Additionally, the miR-29 family can control telomere length by targeting DNA methyltransferases (DNMT). **(D)** Snail, Slug, and Wnt are key factors in epithelial to mesenchymal transition (EMT), which is associated with cancer metastasis and invasion. The diagram shows the related miRNAs. **(E)** Vascular endothelial growth factors (VEGF) play a crucial role in promoting tumor angiogenesis. MiR-126, miR-128, and miR0-200 have been shown to target VEGF. **(F)** Cancer cells use aerobic glycolysis as their main metabolic pathway. For example, lung cancer cells with decreased levels of miR-144 exhibit increased expression of glucose transporter (GLUT1) and higher glucose uptake. **(G)** The interaction between programmed death-ligand 1 (PD-L1) and its receptor (PD-1) allows cancer cells to evade immune destruction. MiR-34, miR-138, miR-200, and miR-513 have been found to target PD-L1 and inhibit its expression. **(H)** Fas receptors (intrinsic pathway) and BH3-only proteins (extrinsic pathway) play important roles in preventing cell apoptosis. MiR-301b targets BH3-only proteins and miR-16 targets Bcl, both of which are involved in regulating cell apoptosis. Note: This diagram simplifies the major pathways involved in cancer, but there may also be cross-talk and interactions between different pathways that are not shown. For example, the EGFR pathway can not only promote cell proliferation but also enhance invasion, metastasis, angiogenesis, and resistance to apoptosis. This figure adapted from (Wu et al., 2019).

Cancer is an immensely complex condition across multiple levels, and its development and behavior require thorough investigation. In 2000, Hanahan et al. identified six significant traits of cancer such as sustaining a proliferation signal, evading growth inhibitors, and activating invasion and metastasis. Ten years down the line, Hanahan and colleagues added two more components to the list: avoiding immune destruction and disrupting cell energy. They have also suggested the aspects of promoting inflammation and genetic instability augment the emergence of tumors (Hanahan and Weinberg, 2011). For understanding cancer biology, both genetic and epigenetic factors must be considered. In this paper, we will discuss microRNAs (miRNAs) and their connected pathways that are involved in each pillar of cancer biology. Figure 1 presents an overview of miRNAs and their relevant pathways.

miRNAs play a role in regulating various genes and signal pathways. A considerable amount of evidence indicates that miRNAs are involved in lung cancer progression, either functioning as tumor suppressors or oncomirs. They exert their effects by modulating the expression of target mRNAs, which in turn impacts cancer biology and facilitates tumor growth, invasion, angiogenesis, and evasion of the immune system. Numerous studies have demonstrated the significance of miRNAs in regulating processes such as cell development, proliferation, invasion, migration, apoptosis, and metastasis in both SCLC and NSCLC (Frydrychowicz et al., 2023). When miRNA expression is deregulated, it leads to changes in their functionality. When miRNAs are overexpressed or underexpressed, they can act as either oncogenes, promoting the development of cancer, or tumor suppressor genes, inhibiting cancer growth. Interestingly, the same miRNA that acts as an oncogene in one type of cancer may have the opposite effect of inducing tumor suppression in another type of cancer. This is because miRNAs have multiple targets and can carry out various biological functions (Iqbal et al., 2019).

miR-21 exerts oncogenic activities by inhibiting the activity of various vital tumor suppressor genes (Far et al., 2022). A study of miRNA expression in various human tumor tissue samples uncovered that miR-21 was the sole miRNA that demonstrated an increment in each of the tumors surveyed (Far et al., 2022). NSCLC patients who show a substantial amount of miR-21 in their systems tend to be at an advanced stage of progression and frequently experience metastasis. Additionally, miR-21 has been demonstrated to accelerate the growth of cells and further a tumor’s invasion by impeding PTEN, a suppressor of tumor cells (Zhang J.-g. et al., 2010; Xue et al., 2016). The expression levels of miR-21 are heightened and linked to worse survival outcomes in NSCLC. miR-21 likely has oncogenic effects in NSCLC, as it has been proven in experiments that it hampers the action of Ras/MEK/ERK signaling mechanism inhibitors (Hatley et al., 2010). This indicates that miR-21 may present potential opportunities for cancer treatment.

Reinhart made the first identification of a 21-nucleotide-long non-coding RNA, namely, let 7, in the context of his research on developmental timing in the nematode *Caenorhabditis elegans* (Reinhart et al., 2000). Research has indicated that the Let-7 gene family consists of 11 members, six of which are situated in areas of the DNA that are sensitive to changes, making them likely to be affected by genetic modifications (Calin et al., 2004). It is possible that the lowering of let-7 levels may be caused by an alteration to its genetic material through epigenetic or genetic pathways, or because of agents that stop its transcribing process (Yamada et al., 2008). Let-7 plays a major part in reducing the activity of proliferation, inflammation, and anti-apoptotic pathways by lessening the activity of their consequent effectors, which include KRAS, c-MYC, CDK6, HOXA9, TGFBR1, BCL-XL, and MAP4K3, and thus producing an anti-cancerous atmosphere inside the cell (Wang X. et al., 2012). Various members of the Let-7 family of genes are transcribed from parts of the genome that are often absent in people with lung cancer, including let-7a, let-7c, and let-7g (Boyerinas et al., 2010). MiR-let-7 has been linked to tumor suppression, as demonstrated by its ability to decrease the expression of KRAS (Slack and Weidhaas, 2006). Recent studies involving the injection of MiR-let-7 mimic into a laboratory model of NSCLC led to a substantial decrease in the tumor area, size, and spread when compared to the control group (Trang et al., 2011). This has led to the suggestion that MiR-let-7 could be a useful treatment technique for lung cancer, having a positive correlation with patient prognosis in lung cancer (Xia et al., 2014).

It is believed that cancer’s core features involve prolonged cell division and unregulated cell growth. This process involves a variety of different genes and proteins, primarily kinases and their associated receptors (Van Roosbroeck and Calin, 2017). One of the most well-known signaling pathways in lung cancer is the Epidermal Growth Factor Receptor (EGFR) pathway. When certain molecules such as EGF or TGF-α bind to EGFR, it is in turn activated and transphosphorylated. This generates additional activation of two significant signaling pathways - Ras/Raf/MEK/ERK and PI3K/Akt/mTOR - leading to an increase in cell growth and an enhanced development of the cell cycle (Brambilla and Gazdar, 2009). It has been established that EGFR has an active association with miR-34, miR-27a-3p, miR-7, miR-30, miR-133, miR-146, miR-145, miR-218, miR-149, miR-128, miR-134, and miR-542-5p (Chan et al., 2012; Qin et al., 2016; Zhu et al., 2016; Van Roosbroeck and Calin, 2017).

The discovery of EML4-ALK fusion proteins as potential therapeutic targets for NSCLC is based on their capacity to activate the Ras/Raf/MEK/ERK and PI3K/Akt/mTOR signaling pathways (Karachaliou and Rosell, 2014). Vishwamitra et al. conducted research on a cell model that revealed that miR-96 acts as a post-transcriptional inhibitor of ALK (Vishwamitra et al., 2012).

The enzymes of glycolysis control the amount of glucose moving into cells, particularly the hexokinases (HKs), which act as gatekeepers by converting glucose to glucose-6-phosphate (G6P) in the initial reaction of glucose entering the metabolic process (Robey and Hay, 2006). HK1 and HK2 are identified as enzymes with a strong affinity, although the amount of these enzymes present can vary throughout different tissues. An unusual amount of HK2 is seen as a sign of malignant tumor formation (Mathupala et al., 2001). Studies using cancer cell lines and mice models as test subjects have shown that higher levels of HK2 are only found in cases of lung cancer (Patra et al., 2013). miR-124 is a microRNA that can impede tumor growth and affect glycolysis, lactate manufacturing, and ATP creation negatively. In lung cancer cells, when the expression of miR-124 is diminished, there is a considerable increment of glucose consumption and ATP production brought about by the bigger expression of GLUT1 and HK2 enzymes, two elements that are critical for controlling the glycolysis pathway (Zhao et al., 2017). The fact that changing the expression of Akt can reverse the effects of inhibiting miR-124 further emphasizes the role of miR-124 in modulating the activity of the AKt1 and AKt2 subunits, as well as regulating glycolysis.

The miR-17/92 family, located at human chromosome 13q31.3 in the intron three region of gene C13orf 25, contains six microRNAs: miR-17, miR-18a, miR-19a, miR-20a, miR-19b-1, and miR-92a-1. This chromosome location is commonly found to be increased in various kinds of tumors (Ota et al., 2004). Hayashita et al. were the first to discover that the miR-17/92 cluster is expressed at higher levels in lung cancer cells and helps to stimulate the expansion of these cells (Hayashita et al., 2005). The inhibition of the miR-17/92 family has a detrimental effect on non-small cell lung cancer cells that lack p53, because of its role in the reduction of CYP24A1 expression (Borkowski et al., 2015). The miR-19 family has been shown to upregulate Wnt signaling through targeting of p38α in non-small cell lung cancer (NSCLC), thus leading to an increased malignant potential in the cell type (Guinot et al., 2016). It has been revealed that the miR-19 family influences the malignant potential of NSCLC cells by increasing Wnt signaling through the blocking of p38α. Additionally, the suppression of miR-19b has been found to lower the phosphorylation of ERK, AKT, and effector proteins in EGFR mutant NSCLC cells. This means that targeting miR-19b could be a viable option for the treatment of EGFR mutant NSCLC (Baumgartner et al., 2018).

The KRAS gene is often linked to receptor tyrosine kinases such as EGFR, ALK, and ROS. Kirsten rat sarcoma 2 viral oncogene homolog is the specific name for this gene (Friday and Adjei, 2005). Approximately one-sixth of lung cancer specimens contain at least one KRAS mutation, which can prompt the Ras/Raf/MEK/ERK pathway to become active (Kopp et al., 2014). In laboratory and mouse testing, the let-7 family showed its ability to control KRAS expression and suppress the growth of lung cancer cells (He et al., 2010; Wang Y. Y. et al., 2013). Seviour and their team’s investigation uncovered that miR-193a-3p has a direct influence on KRAS, thereby inhibiting the growth of KRAS-mutated lung tumors in a living organism (Seviour et al., 2017). Given that miR-181a-5p is known to inhibit the activity of KRAS, it appears that this molecule may play a role in the progression and movement of A549 cells (Ma et al., 2015). Research has shown that miR-148a-3p can limit the development of non-small cell lung cancer cells in the lab through the regulation of SOS2, a chemical found in the Ras signaling pathway’s chain of events (Xie et al., 2019). Jiang et al. also found that miR-1258 could influenc GRB2 resulting in lower Grb2 levels, which was a fundamental protein needed to carry on Ras activation, consequently lowering the MEK/ERK pathway in mouse models (Jiang et al., 2018). The research conducted in a lab indicated that the JmiR-520a-3p participated in a downstream PI3K/Akt/mTOR signaling pathway (Lv et al., 2018). Table 1, lists various miRNAs that are involved in lung cancer.

**TABLE 1:** Various miRNAs in lung cancer.

MicroRNA	Expression	Target	Method	Cell line	Ref
Let-7a	Down	KRAS	*In-vivo*	-	Pulliero et al. (2023)
Mir-185-5p	Down	YWHAZ	*In-vivo, in-vitro, human*	NCl-H322, A549, NCl-H1299, PC9, BEAS-2B	Ma et al. (2023)
miR-452-5p	Up	Moesin	*In-vitro*	H1703, H1299, A549, H460, H322, HNBE	Zhuang et al. (2023)
miR-375	Up	ERK	*In-vivo, in-vitro, human*	HBE, NCI-H520, NCI-H226, NCI-H2170, SK-MES-1, HEK-293	Gan et al. (2023)
miR-34c-5p	Down	Snail1	*In-vivo, in-vitro, human*	A549, H1299	Yang et al. (2023a)
MiR-503	Down	PTK7	*In-vivo, in-vitro*	HCC827, CL1‐5, H1299, 293-T	Tsai et al. (2023)
miR-183-5p	Down	LOXL4	*In-vitro, human*	A549, 95D, H1299, H1650	Chen et al. (2023a)
miR-494	Up	PUMA-α	*In-vitro, human*	NCI-H520, SW900, EBC-1, SK-MES-1	Gao et al. (2023)
miR-219-5p	-	-	*human*	-	Wu et al. (2023a)
miR-508-5p	Down	AKT	*In-vitro, human*	NCI-H1395, SPC-A1, A549, Calu-3, BEAS-2B, HEK293T	Wu et al. (2023b)
miR-26a-5p	-	POLR3G	*In-vitro*	H23, H358, H226, H460, H1299, Hcc1438	Park et al. (2023)
miR-146b-3p, −146b-5p	-	-	*in-vivo, in-vitro*	A549, BEAS-2B	Patnaik et al. (2011)
miR-423-3p	Up	-	*Human, in-vitro*	A549, H1299, HCC827, A427, BEAS-2B	Wang et al. (2019a)
miR-654-3p	Down	RASAL2	*Human, in-vitro*	A549	Xiong et al. (2021)
miR-1-3p	-	FAM83A	*In-vitro*	A549, H1299	Liu et al. (2020a)
miR-16	Down	MEK1	*In-vitro*	Anip973, AGZY83-a, BEAS-2B	Chen et al. (2019a)
miR-141	Up	KLF9	*Human, in-vitro*	A549, H460, MRC-5	Kong et al. (2019)
miR-206	Up	-	*Human, in-vitro*	SPC-A-1, A549, 95D, LTEP-Sm1, NCI-H226, NCI-H520	Wang et al. (2011)
miR-27b	Down	Snail	*Human, in-vitro*	A549, H1299	Zhang et al. (2020a)
miR-16	Down	YAP1	*In-vitro*	SK-MES-1, A549, MS-53, SK-LU-1	Wei et al. (2020)
miR-151a-5p, miR-23b	Up	-	*Human, in-vitro*	A549	Guo et al. (2020a)
miR‐200c‐3p, miR‐485‐5p	-	-	*Human, in-vitro*	A549, H1299, BEAS‐2B	Liu et al. (2022a)
miR-663	Up	TGFB1	*In-vitro*	A549	Liu et al. (2011)
miR-148a	Down	-	*Human, in-vivo, in-vitro*	SPC-A-1, A549, H1299 LC-2, H358	Li et al. (2015)
miR-203	-	PKCα	*Human, in-vitro*	A549	Wang et al. (2013b)
miR-185	Down	-	*Human*	-	Liu et al. (2020b)
miR-186	Down	-	*Human*	-	Xie et al. (2020)
miR-340-5p	Down	ZNF503	*Human, in-vitro*	BEAS-2B, A549, NCI-H460, NCI-H1299, NCI-H1650, NCI-H292	Lu and Zhang (2019)
miR-25	Up	cyclin E2	*Human, in-vitro*	H146, H209, G446, H510, H889, MRC5	Zhao et al. (2014)
miR-138	Down	SOX4	*In-vitro*	MRC-5, HCC827, A549, SK-LU-1, A427	Xing et al. (2020)
miR-34c	Down	NOTCH1	*In-vitro*	A549, H1299, 293T	Yang et al. (2020a)
miR-1, miR-133	Down	-	*Human*	-	Kazempour Dizaji et al. (2022)
miR-191, miR-24	Up	-	*Human*	-	Kazempour Dizaji et al. (2022)
miR-129b	Down	-	*Human, in-vitro*	A549, H1299	Zheng et al. (2016)
miR-616	Up	SOX7	*In-vivo, in-vitro*	H-358, H-1703, A549, NL-20	Wang et al. (2017)
MicroRNA-29a	Down	NRAS	*In-vitro*	H1299, A549, HEK293 T	Liu et al. (2018)
miR-218	Down	Robo1	*Human, in-vitro*	A549, HCC4006	Chen et al. (2017a)
miR-140-3p	Down	ATP6AP2	*Human, in-vitro*	A549, H1299	Kong et al. (2015)
miR-133b	Down	FOXL2	*Human, in-vitro*	A549, H460, SPC‐A1, H1299, H1650, H1975, PC‐9, BEAS‐2B	Li et al. (2023a)
miR-152-3p	Down	NCAM1	*In-vivo, in-vitro, human*	A549 and H446	Zhao et al. (2023a)
miR-214	-	β-catenin	*Human, in-vitro*	H1299, SPC, A549, H157, HBE	Zhao et al. (2023b)

## Long non-coding RNAs and lung cancer

LncRNAs, which make up 81.8% of all ncRNAs, are the most abundant type of ncRNA. They demonstrate distinct patterns in terms of their origins, timing, and location within specific tissues and cells, although they are not as abundant, stable, or conserved as mRNA. These crucial molecules play a role in regulating nearly every aspect of gene expression (Liu et al., 2021). The primary method in which they operate is to block the expression of target genes by attaching to miRNA and implementing an additional level of post-transcriptional regulation. In addition, lncRNAs have the ability to divert transcription factors (TFs) away from chromatin as a means of acting as molecular reservoirs, effectively changing the expression of genes. Furthermore, evidence suggests that they act as building blocks to form scaffolding complexes with regulators, ultimately leading to modifications in gene expression. Additionally, lncRNAs can direct the coordination of ribonucleoprotein to the promoters of downstream target genes, ultimately altering the transcriptional activity of genes. In addition, lncRNAs have the capacity to modify gene expression by influencing the processing, maturation, and stability of mRNAs (Lv et al., 2023).

**FIGURE 2:**
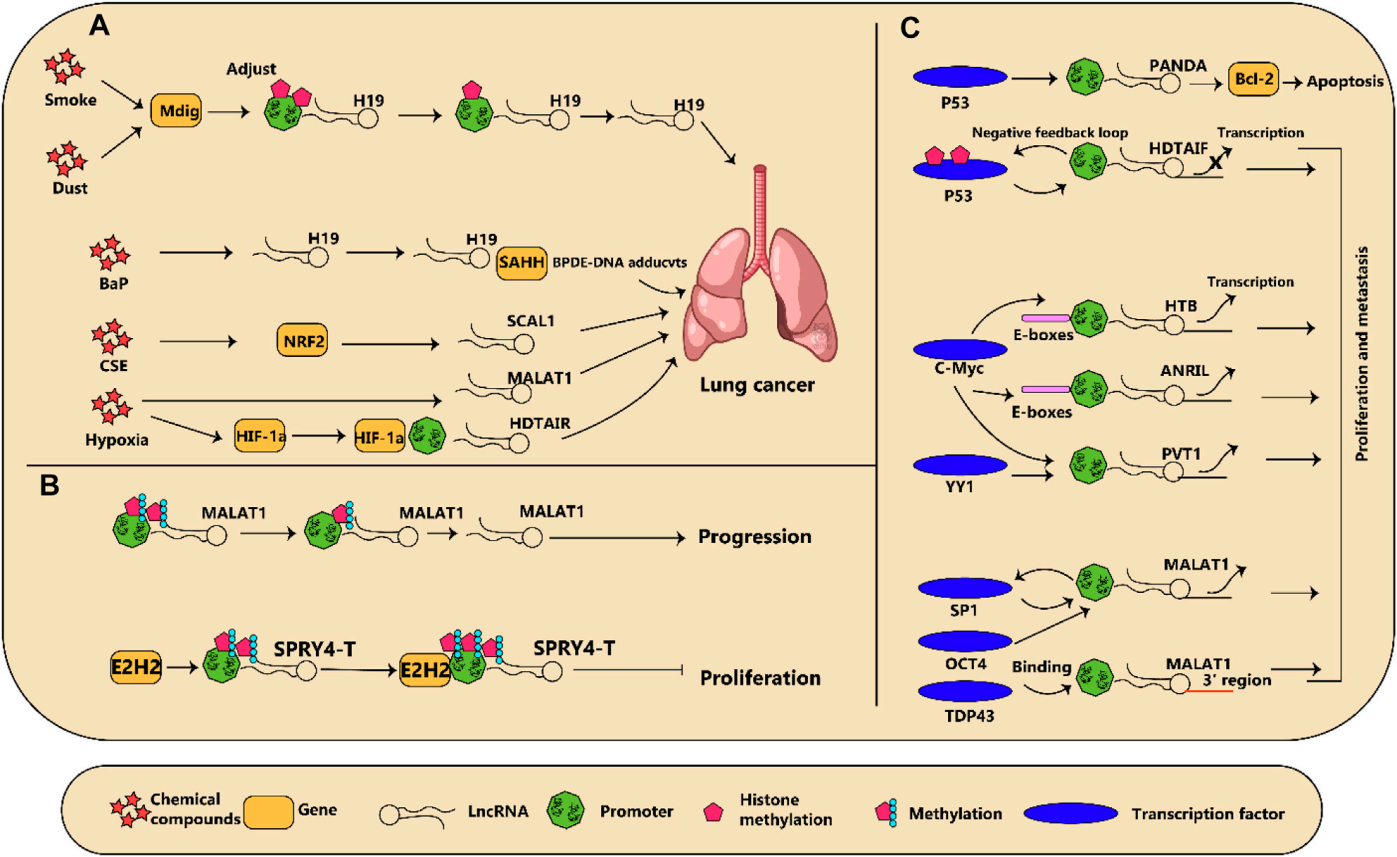
**(A)** diagram illustrating how regulation of dysregulated long non-coding RNAs (lncRNA) in lung cancer is influenced by various factors is shown. **(A)** Chemical compounds and reduction in oxygen levels regulate lncRNA expression which subsequently results in the advancement of cancer. **(B)** Modifications to the epigenetic makeup of lncRNAs can impact the growth of lung tumors. **(C)** Several transcription factors connect to lncRNAs and either incite or discourage their transcription, leading to changes in cancer development.

Many research projects have used microarray profiling and deep sequencing data to demonstrate that an alteration in lncRNA expression is a primary component of both the initiation and progression of lung cancer (Gencel-Augusto et al., 2023; Rajakumar et al., 2023; Tang et al., 2023). There are specific mechanisms that can affect their expression in pathological conditions, which include the influence of chemicals and microenvironments in tumors. Furthermore, gene expression can be modified without any changes to the content of the DNA through epigenetic modification. The binding of transcription factors on the promoters of lncRNAs can trigger either their activation or their suppression; these various regulation models of lncRNAs in lung cancer have been summarized in Figure 2 and Table 2.

**TABLE 2:** Various lncRNAs in lung cancer.

LncRNAs	Expression	Target	Method	Cell line	Ref
SOX2	Up	miR-122-3p, miR-194-5p	*In-vitro*	A549, Calu-3	Dodangeh et al. (2023)
lnRNA-ICL	Down	miR19-3p	*In-vivo, in-vitro, human*	Primary LC cells	Lu et al. (2023)
MCF2L-AS1	Up	miR-33a-5p	*In-vivo, in-vitro, human*	MHCC97H, HCCLM3, 293T	Ou et al. (2023)
ASBEL, Erbb4-IR	-	miR-21	*In-vitro, human*	H226, H2170, 16HBE	Liang et al. (2023)
LINC00638	Up	miR-541-3p	*In-vivo, in-vitro, human*	BEAS-2, BHCC-827, A549, NCI–H460, H1975, H1299, H460	Zhang et al. (2023a)
PSMA3-AS1	Up	miR-329-3p	*In-vivo, in-vitro, human*	GES-1HGC-27, AGS, NCI-N87, SNU-1, HEK-293T	Kan et al. (2023)
TFAP2A-AS1	Up	miR-584-3p	*In-vivo, in-vitro, human*	BEAS-2B, A549, H1299, H460, SK-MES-1	Zhang et al. (2021a)
ITGB2-AS1	Up	FOSL2	*In-vivo, in-vitro, human*	BEAS−2B, SK-MES−1, NCI-H520, Calu−1, LUAD, A549, H1975, PC-9, 293T	Chen et al. (2023b)
LINC01124	Up	miR-1247-5p	*In-vivo, in-vitro, human*	THLE-2, SNU-182, Hep3B, Huh-7	Sun et al. (2021)
PRRT3-AS1	-	miR-507	*In-vivo, in-vitro, human*	A549, H460, SK-MES-1, H1299	Zhou et al. (2021a)
LINC00621	-	TGF-β	*In-vivo, in-vitro, human*	A549, HCC827	Wei et al. (2023)
miR-210HG	Up	MiR-210	*In-vitro, human*	H1650	Cao et al. (2023)
LINC00461	Up	miR-4478	*Human, in-vitro*	16HBE, A549, H1299, H23, SPC-A1	Meng et al. (2020)
lncRNA DSCAS	Up	miR-646-3p	*In-vitro, human*	NHBE, H520, SK-MES-1	Liu et al. (2023a)
LINC00607	Down	miR-1289	*In-vitro, human*	A549, H460, H1299, Beas-2B	Zhang et al. (2023b)
LINC00943	Up	miR-1252-5p	*In-vitro, human*	A549, H1299, H1975, BEAS-2B	Liu et al. (2023b)
LncRNA AK001796	Up	miR-150	*In-vivo, in-vitro, human*	HepG2, SMMC-7721, HUH7, BEL-7402, L02	Xu et al. (2023)
lncRNA PCBP1-AS1	-	ITGAL	*Human*	-	Wang et al. (2023a)
LINC02159	Up	ALYREF	*In-vivo, in-vitro, human*	A549, H1299, PC9HBE	Yang et al. (2023b)
LINC00969	Up	NLRP	*In-vivo, in-vitro, human*	PC9, HCC827 A549, SPCA1	Dai et al. (2023)
HOXD-AS2	Up	miR-3681-5p	*In-vivo, in-vitro, human*	H1975, H1299, BEAS-2B	Zhang and Ma (2023)
DINO	Down	p53-Bax	*In-vivo, in-vitro*	H460, A549, H1299, H1993, Hcc827, PC9, and H1975HBE	Liu et al. (2023c)
POU6F2-AS2	-	miR-125b-5p	*In-vivo, in-vitro, human*	BEAS-2B, A549, SK-MES-1, H460	Yang et al. (2023c)
DGUOK-AS1	Up	TRPM7	*In-vivo, in-vitro, human*	NCI-H520, SK-MES-1, Calu-1, PC-9, A549, BEAS-2B	Feng et al. (2023)
SNHG7	Up	LC3B	*In-vivo, in-vitro, human*	A549, HCC827	She et al. (2023)
LINC00115	Up	miR-154-3p	*In-vivo, in-vitro*	A549, NCI–H1975, NCI–H1299, PG49, BEAS-2B, NCI–H460	Sun et al. (2023a)
LINC01798	-	miR-17-5p	*in-vitro, human*	A549, PC9, H1975, BEAS-2B	Li et al. (2023b)
LANCL1-AS1	Down	miR-3680-3p	*In-vivo, in-vitro*	HBEA549, H1299, H460	Pan et al. (2023)
MALAT1	-	miR-328	*in-vitro, human*	A549/H460	Liu et al. (2023d)
FOXD2-AS1	Up	-	*in-vitro, human*	A549	Yuan et al. (2023)
BC009639	Up	IMPAD1	*In-vivo, in-vitro, human*	BEAS‐2B, PG‐LH7, PG‐BE1, 95C, 95D, the H460, A549	Chen et al. (2023c)
Linc00173	Down	miR-1275	*In-vivo, in-vitro, human*	H1299, H1650, H1975, A549, SPCA1, PC9	Tao et al. (2023)
LINC00669	Up	Wnt/β-catenin	*in-vitro, human*	H1975, H1299, H358, A549, HBE	Zhu et al. (2023a)
LOC285758	Up	miR-204	*in-vitro, human*	BEAS-2B, H292, A549	Yu et al. (2022)
LINC01833	Up	-	*Human*	-	Liu et al. (2023e)
SNHG6	Up	p27	*In-vivo, in-vitro*	A549, SPCA1, H1299, H1975, PC9, 16HBE	Wang et al. (2022a)
A2M-AS1	Down	miR-587	*In-vivo, in-vitro, human*	Beas-2B, H1975, HCC827	Guo et al. (2022)
LINC01635	Up	miR-455-5p	*In-vivo, in-vitro, human*	A549, H1299, H1975, PC9, HBE135	Shen et al. (2022)
KCNQ1OT1	Up	miR-491-5p	*in-vitro*	SK-MES-1, NCI-H226, HEK293	Liu et al. (2022b)
LINC02389	Up	miR-7-5p	*in-vitro, human*	A549, HCC827, MRC-5	Ma et al. (2022)

**FIGURE 3:**
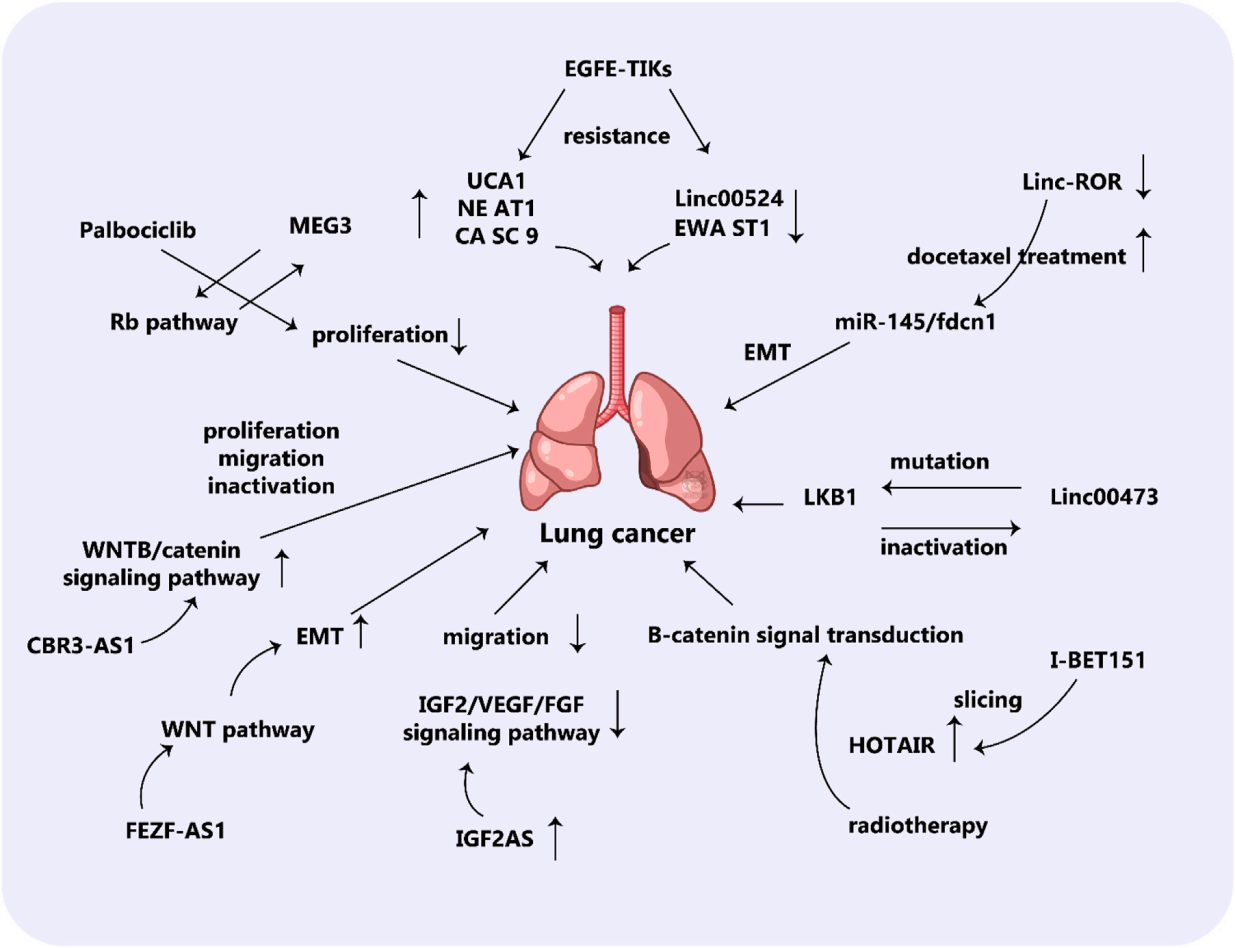
The role of lncRNAs in lung cancer. The lncRNA XLOC_008466 exhibits a high level of expression in patients with NSCLC. When XLOC_008466 expression is suppressed, there is a decrease in cell proliferation and invasion, along with a promotion of apoptosis. This lncRNA acts in a manner similar to a ceRNA, directly binding to and downregulating miR-874. As a result, the expression of miR-874 downstream targets, MMP2 and XIAP, is increased. Additionally, there is an axis involving lncRNA Gm15290, miR-615-5p, and targeted genes in lung cancer. AS5 expression is downregulated in lung cancer tissues and cells, while miR-135b expression is upregulated. The high expression of GAS5 and low expression of miR-135b have been shown to significantly reduce the survival rate of lung cancer cells under irradiation and improve radiotherapy sensitivity. Furthermore, this can also effectively inhibit tumor occurrence by suppressing the proliferation and invasion of tumor cells.

Research has demonstrated that PM2.5 could make people more susceptible to developing lung cancer. It appears lncRNA is involved in this process, as there is an increase in Reactive Oxygen Species (ROS) resulting from large exposure and an increase in lncRNA expression in NSCLC cells. This could also be affected by the autophagy of cancer cells, boosting the infiltration and migration of tumors. Additionally, employment-related exposure greatly affects the rate of occurrence of NSCLC, such as a decrease in the activity of the lncRNA MEG3 – which has anti-growth characteristics – when exposed to nickel (Zhang X. et al., 2010; Wang P. et al., 2012; Lu et al., 2013; Mondal et al., 2015; Zhou et al., 2017). Nickel exposure can lead to hypermethylation and inhibition of the MEG3 promoter, which can eventually cause carcinogenic effects on human bronchial epithelial cells. This research shows just how influential long non-coding RNAs are in the incidence of Non-Small Cell Lung Cancer. Understanding these factors and pathways should help ascertain the biological basis of NSCLC and pave the way to its prevention and treatment (Figure 3). CDKs and CKIs are essential for keeping the cell cycle in balance, an occurrence that is common in cancer. For this, lncRNA lnc00152 and lnc00511, both observed to be increased in LUAD cases, are capable of promoting tumor growth by preventing the activity of IL24 and p57, which are components of the tumor suppressor mechanism respectively (Sun et al., 2016; Chen Q. N. et al., 2017). According to recent research, LUADT1, a long noncoding RNA that has been observed to be significantly expressed in lung adenocarcinoma tumors, is associated with histological T stage. When LUADT1 joins with SUZ12, the trimethylation of H3K27 is cause in the promoter area of the antitumor P27. As a result, this hinders the expression of P27, thus affecting its scope of action epigenetically (Qiu et al., 2015).

It is believed that Cancer stem cells (CSCs) represent a group of tumor cells which display characteristics such as the capacity to renew themselves or to transform into non-stem cancerous progeny. Therefore, these cells tend to play a major role in various functions such as resistance to therapy, development of tumors, change from epithelial to mesenchymal cells (EMT) and metastatic spread of tumors (Clarke and Fuller, 2006; Zhao, 2016). The expression of certain cell surface biomarkers such as CD44, CD133, OCT-4, Bmi-1, ALDH1, ABCG2 and KLF4 is highly characteristic of cancer stem cells (CSCs), and is thought to be linked to the decreased susceptibility of CSCs to cancer treatments. For instance, previous studies have investigated the characteristics of CSCs resistance to cancer treatment, including slower-than-normal cell division rates and dormant states (Vidal et al., 2014; Ajani et al., 2015). The cancer microenvironment, along with multiple regulatory factors, are integral to both the maintenance of cancer stem cells (CSCs) and the growth and advancement of tumors. Recently, lncRNAs that are involved in both CSCs and tumor evolution have been the focus of many studies (Schwerdtfeger et al., 2021).

LnRNA HOTAIR has been observed to activate tumor growth through its influence on CSCs (cancer stem cells) and EMT processes, and its activity is triggered directly by STAT3 (signal transducer and activator of transcription 3) when a person is exposed to cigarette smoke (Liu et al., 2015). Previous research has evaluated the connection between being a stem cell and EMT pathways (Wilson et al., 2020). The influence of DUXAP10 increased in Cd-induced lung cells, while eliminating DUXAP10 caused a decrease in stemness markers like KLF4, KLF5 and Nanog. Moreover, this led to a reduction in the number of stem cells labeled by CD133, which was due to the inhibition of the Hedgehog signaling pathway which played a part in Cd-induced cancer of the lung (Lin et al., 2021). Liu et al. demonstrated that HOTAIR, a long non-coding RNA, could generate resistance to cancer treatments such as cisplatin by prompting two stem cell markers - β-catenin and KLF4, with an emphasis on specifically influencing KLF4 to enhance stemness (Liu M. Y. et al., 2016).

**FIGURE 4:**
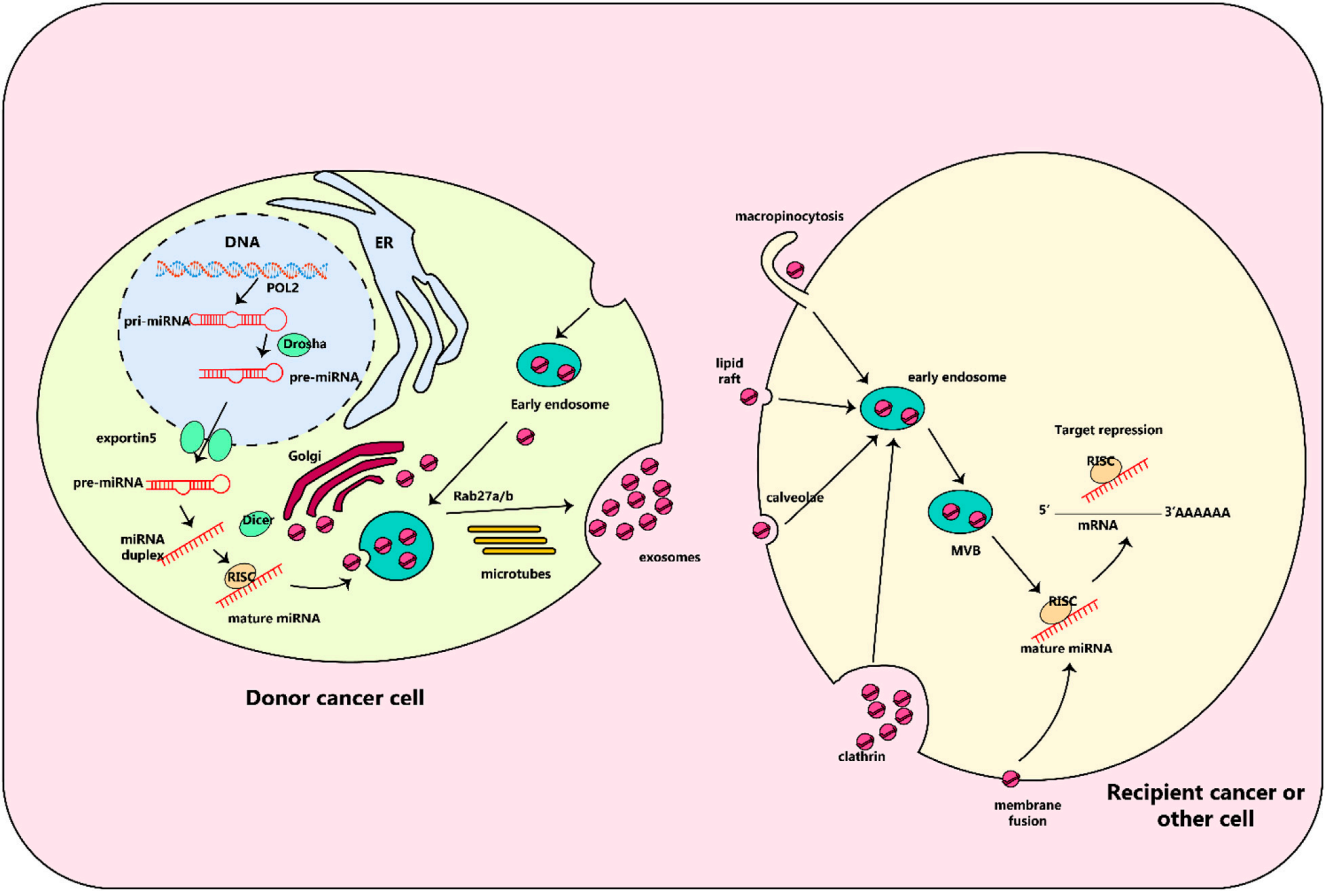
Biology of exosomal miRNAs. In animals, RNA polymerase II transcribes microRNA (miRNA) genes, producing primary miRNAs (pri-miRNAs), which are then modified by the Drosha complex into precursor miRNAs (pre-miRNAs). These pre-miRNAs are then transported to the cytoplasm by the exportin5 complex. After being digested by the Dicer complex, they become double-stranded miRNAs and are converted into single-stranded mature miRNAs with the help of a helicase. These mature miRNAs are then sorted into multivesicular bodies (MVBs), which are transported along microtubules to the plasma membrane and released as exosomes. These exosomes, containing specific miRNAs from the parent cell, can interact with recipient cells through various mechanisms including fusion through clathrin-dependent or independent endocytosis, caveolae-mediated endocytosis, or lipid raft-dependent endocytosis. Once inside the recipient cell, the exosomal miRNAs may repress their target genes.

Xue et al. found that GAS5 levels were lower and miR-135b levels higher in NSCLC tissues and cells (Xue et al., 2017). The analysis discovered that non-small cell lung cancer cells were more sensitive to the effects of radiation when there was an elevated presence of GAS5 and a depleted presence of miR-135b, making radiation treatments more efficient. This was seen *via* decreased survival rates when the cells were exposed to radiation, and decreased spread and increased deterrence of tumor growth due to more restricted tumor cell proliferation and invasion (illustrated in Figure 4).

The lncRNA MALAT1 has a length of 8.7 kb, and is commonly found in human tissue types. Overall, it exhibits conservation across mammalian species (Zhang et al., 2017). The dysregulation of MALAT1 has been observed in different types of cancers such as lung, liver, prostate, colon, uterus, ovarian, breast, neuroblastoma, and blood-based cancers. Its role appears to involve the post-transcriptional control of the gene expression and splicing of mRNA (Bhan et al., 2017; Huang et al., 2017; Li Z. X. et al., 2018; He et al., 2019; Sun and Ma, 2019).

Malat1 is an oncogenic lncRNA that can increase cancer cell proliferation, migration, invasion, and EMT. Thus, Malat1 may be responsible for cancer cell survival and growth. Malat1 may also play a role in cancer chemoresistance (Schmidt et al., 2011; Li Z. X. et al., 2018; Sun and Ma, 2019). It seems that MALAT1 is more highly expressed in NSCLC tissues than in normal tissues and that MALAT1 expression is correlated with the overall survival of NSCLC. If this is true, then MALAT1 may be a useful biomarker for identifying patients who are likely to benefit from treatment with chemotherapy (Chen et al., 2018). Some research suggests that MALAT1 might play a role in regulating the myeloid-derived suppressor cells (MDSCs) in lung cancer patients. This could potentially lead to problems with the immune system’s ability to fight cancer (Zhou et al., 2018). Silencing of MALAT1 has been shown in cultured NSCLC cells to decrease proliferation and colony formation (Gutschner et al., 2013).

MALAT1 may also act as a regulator of several important genes by way of a non-coding RNA-mediated process. For example, MALAT1 can increase the expression of zinc finger E-box binding homeobox 1 (ZEB1) in A549 cells by rapidly soaking up miR-200a, thus stimulating cell proliferation (Feng et al., 2019). The miR-200b protein plays a role in linking up with the E2F transcription factor 3 (E2F3) and ZEB1 mRNAs which are found in the cytoplasm of the cells. When miR-200b interacts with these mRNAs, it can lead to the levels of E2F3 and ZEB1 proteins being raised in DTX (docetaxel) - resistant lung adenocarcinoma cells. This then allows these cells to become more tolerant of chemotherapy. Therefore, miR-200a could be proposed as a potential prognostic marker to identify DTX–resistant patients (Chen J. et al., 2019). The effects of MALAT1 on the regulation of different processes are outlined in Figure 4.

## Circular RNAs and lung cancer

CircRNAs are non-coding RNA molecules that originate from the back-splicing of pre-mRNA and have a closed-loop structure. This configuration allows them to resist degradation by exonucleases, enabling their presence in different subcellular compartments. Due to their involvement in critical biological activities such as gene regulation, protein production, immune response, and cancer development, circRNAs play a significant role in numerous cellular functions and contribute to drug resistance (Wang et al., 2020a). The main focus of research on circRNAs is their ability to diminish the impact on target mRNAs by acting as miRNA decoys, ultimately leading to changes in correlated gene expression. These circRNAs typically contain numerous miRNA binding sites. Additionally, circRNAs play a role in various biological processes by modulating the functions of proteins (Zhang et al., 2021b). Moreover, studies have revealed that EIciRNAs play a role in promoting the transcription of their source genes by interacting with U1 small nuclear ribonucleoproteins and facilitating RNA polymerase II activity. Additionally, it has been shown that CircURI1 plays a crucial role in regulating alternative splicing of numerous genes associated with cell migration by directly binding with hnRNPM, ultimately suppressing the metastatic progression of gastric cancer. Furthermore, some endogenous circRNAs containing open reading frames have been observed to undergo translation, resulting in the production of peptides or proteins (Shoda et al., 2022). However, their potential functions are still unclear.

Much interest has arisen with regard to circRNAs potentially having a role in tumor formation and tumor evolution. Research conducted on certain forms of lung cancer has reaffirmed the crucial role circRNAs play in the disease (Gao L. et al., 2020; Pandey et al., 2020). The main ideas with regard to tumorigenic mechanisms revolve around the disruption of the normal cell cycle and the emergence of greater proliferative power. The dynamic of CDKs and cyclins during this process has been determined to be a critical factor in the regulation of the cell cycle (Arcinas et al., 2019). As of late, it has been identified that certain circRNAs may support the biological activities of cyclin-dependent kinases (CDKs) or cyclins in lung cancer (You et al., 2015). As an illustration, circPVT1 interferes with the expression of miR-30d and miR-30e to quicken the transcription of cyclin F mRNA, causing the increased production of cyclin F that encourages the advancement of LUSC cells (Stoll et al., 2020). In contrast, circRNA cESRP1 impacts the cell cycle by attaching to miR-93–5p; this in turn promotes the activity of p21, thereby blocking the activity of CDKs (Zhao Q. et al., 2020). Additionally, an earlier investigation found that circRACGAP1 preserved the growth of NSCLC cells through its capacity to absorb miR-144–5p; this also enabled circRNA to directly bind linear mRNA, resulting in the modulation of CDKL1 expression (Hanan et al., 2017).

An array of data suggests that aberrant expression of circRNAs may participate in the development and spread of lung cancer. Evidence points to circPTK2 which appears to interact with miR-429/miR-200b-3p to boost EMT through TIF1γ, posing a higher risk of NSCLC cells (Zhang H. et al., 2018).

The expression of Circ-ABCB10 is augmented in non-small cell lung cancer (NSCLC) cell lines, and knocking down the circ-ABCB10 increases microRNA miR-1252 while reducing levels of Forkhead box 2 (FOXR2), resulting in decreased migration of the NSCLC cells (Huang W. et al., 2020). The Circ-MAN2B2 gene has been shown to increase lung cancer cell invasion through its activation of the miR-1275/FOXK1 pathway (Qu et al., 2018). Circ-0067934 has been reported to have elevated expression concerning the capacity of cancer cells to migrate and infiltrate, together with increased expression of several proteins related to Epithelial-Mesenchymal Transition (EMT) processes, such as N-cadherin (Xue et al., 2019). Circular RNA cESRP1 acts as a sponge for miR-93-5p, leading to reduced levels of Smad 7/p21(CDKN1A), thus preventing TGF-β-driven EMT from progressing in lung cancer (Wei et al., 2019). Circ-BANP amplifies the mobility, incursion, and augmented manifestation of LARP1 in lung cancer cells using the enhancement of miR-93-5p and the decrease of miR-503 (Liu G. et al., 2019). The level of the circulatory gene CircAGFG1 is higher in Non-Small Cell Lung Cancer cells and it increases the process of invasion, migration, and switching between epithelial and mesenchymal forms through the combined action of circ-AGFG1, miR-203, and ZNF281 (Tan et al., 2020). It appears that Circ-PVT1 has a marked presence in LUAC cell lines and tissues, indicating its correlation with CDDP and MTA. It is speculated that Circ PVT1 is responsible for transporting resistance to CDDP and MTA through the miR-145-5p/ABCC1 axis and that its deficiency may magnify tumor cells’ susceptibility to these chemotherapeutics (Mao and Xu, 2020). Mao and his colleagues showed that CDR1-AS is also increased in LUAC tissues and cells, promoting patient immunity to PTX and CDDP using the EGFR/PI3K path (Kong, 2020). The expression of cESRP1 is considerably lower in chemoresistant cells, which increases the cells’ sensitivity to drugs by interacting with miR-93-5p in small cell lung cancer, thereby raising the levels of CDKN1A and averting the transformations caused by transforming growth factor-β using miR-93-5p (Wei et al., 2019).

MAPK pathways are vital for regulating diverse cellular activities, including the stimulation of cell proliferation, differentiation, and cell death (Fang and Richardson, 2005; Guo Y. J. et al., 2020). These pathways involve the MAPK/ERK family, BMK-1, JNK, and p38 pathways as part of the classical route (Sun et al., 2015). Chen et al. identified that hsa_circ_0007580 was an increased circRNA in lung cancer and affected the MAPK signaling pathways. This complex mechanism instigated miR-545-3P to produce a sponge effect on PKCA, thus stimulating NSCLC cell growth and invasion by igniting the p38/MAPK signaling pathway (Chen et al., 2020). In a xenograft tumor model, hsa_circ_0007580 which had a lower expression level impeded the growth of NSCLC tumors by switching off the p38/MAPK pathway. Additionally, circ_0074027 augmented the malignant character of NSCLC cells by absorbing miR‐185‐3p and amplifying the amount of bromodomain‐containing protein 4 (BRD4) and MAPK‐activating death domain-containing protein (MADD) (Gao P. et al., 2020). A study conducted by Zhang and their colleagues determined that hsa_circRNA_101237 enhanced MAPK1 expression through its association with miRNA-490-3p, which then had a direct influence on the proliferation, migration, and invasion of non-small cell lung cancer cells. This is indicative of hsa_circRNA_101237s role as a significant one-circRNA (Zhang Z. Y. et al., 2020). Wang et al. observed that circ-ZKSCAN1 can absorb the cancer-causing miR-330-5p, leading to an increase in Family with Sequence Similarity 83 (FAM83)-member A, hindering the MAPK signal transduction pathway and stimulating non-small cell lung cancer (Wang Y. et al., 2019). There was a great reduction in the amount of JNK, p38, and ERK when there was an excessive amount of circZKSCAN1 present in NSCLC cells (Wang Y. et al., 2019). The results suggested that, in addition to circ-ZKSCAN1, which inhibited MAPK signal transduction and had a carcinogenic effect, other circRNAs could possibly promote lung cancer growth through the activation of MAPK signaling.

The generation of circular protein kinase C iota (CircPRKCI) from exons 15 and 16 of the PRKCI gene (chr3:170013698-170015181) at the 3q26.2 amplicon has been linked to tumor progression in lung adenocarcinoma (LAD). Studies have produced evidence of a positive correlation between the presence of CircPRKCI and high T stage and TNM stage in LAD patients (Qiu et al., 2018). The efficacy of circPRKCI in therapy was tested using the PDTX model in nude mice. This was done by introducing cholesterol-conjugated si-circPRKCI *via* an intratumoral injection and observing the consequences. There was a decrease in tumor size and mass in the si-circPRKCI group compared to the control group. This finding brings into focus the potential of circPRKCI for the treatment of tumors (Qiu et al., 2018). In the study, researchers looked at how effective different combinations of EGFR-TKIs and knockdown of circPRKCI (a protein that regulates EGFR) were in treating NSCLC patients. They found that the combination of EGFR-TKIs and knockdown of circPRKCI had a more notable inhibitory effect on cancer progression than gefitinib or knockdown of circPRKCI alone. This suggests that a combination of EGFR-TKIs and attenuation of circPRKCI may have a synergistic effect on reducing cancer progression (Qiu et al., 2018). Table 3, lists various circular RNAs that are involved in lung cancer.

**TABLE 3:** Various circular RNAs in lung cancer.

CircRNAs	Expression	Target	Method	Cell line	Ref
circ_0000376	Up	miR-1298-5p	*In-vivo, in-vitro, human*	A549, H1299	Hu et al. (2023a)
circ_0010235	Up	miR-34a-5p	*In-vivo, in-vitro, human*	H1299, A54HBE	Zhang et al. (2023c)
Circ-PDZD8	Up	miR-330-5p	*In-vivo, in-vitro, human*	A549, H520, BEAS‐2B	Zhu et al. (2023b)
Circ_0010235	Up	miR-379-5p	*In-vivo, in-vitro, human*	HBE, A549, H1299	Wang et al. (2023b)
circCCDC134	Up	miR-625-5p	*In-vivo, in-vitro, human*	H1299, HCC827, PC9, A549, BEAS‐2B	Tong et al. (2023)
circ_007208	Up	miR-1225-5p	*In-vivo, in-vitro, human*	16-HBE, H1299	Zhu et al. (2023c)
circ-ANXA7	Up	miR-545-3p	*In-vivo, in-vitro, human*	A549, H460, HBE-1	Yao et al. (2023)
Circ_0087378	-	miR-199a-5p	*In-vitro*	16HBE, A549, NCI-H1299, NCI-H1975, HCC827, PC9	Ming et al. (2023)
circ-YES1	Up	miR-142-3p-HMGB1	*In-vivo, in-vitro*	BEAS-2B, H1650, A549, PC-9	Jin et al. (2023)
Circ_0003028	Up	miR-1305	*In-vitro, human*	A549, HCC827	Shi et al. (2023)
circTADA2A	-	miR-455-3p	*Human, in-vivo, in-vitro*	A549, H1299	Zhao et al. (2020b)
circ_0006423	Down	miR-492	*Human, in-vitro*	A549, NCI-H1299, NCI-H1573, BEAS-2B, A549, NCI-H1299	Zhu et al. (2022a)
circ_0072309	Up	miR-100	*Human, in-vivo, in-vitro*	A549	Zhang et al. (2023d)
circ_0003220	Up	miR-489-3p	*Human, in-vitro*	293T, H460, A549, HBE	Xia and Wang (2023)
ZKSCAN1	Up	miR-185-5p	*In-vitro, human*	A549 and PC‐9HBE1	Yu et al. (2023)
circ_PLXND1	Up	miR-1287-5p	*Human, in-vivo, in-vitro*	16HBE, H1299	Wu et al. (2023c)
circDLG1	Up	miR-144	*Human, in-vivo, in-vitro*	CALU3, SPCA1, A549, H1229, 16HBE	Chen and Xu (2023)
circ_0000376	Up	miR-545-3p	*Human, in-vivo, in-vitro*	16HBE, H522, A549	Sun et al. (2023b)
circFBXO7	Down	miR-296-3p	*Human, in-vivo, in-vitro*	A549, H226	Wang et al. (2023c)
circ_0070659	Up	miR-377	*In-vitro, human*	A549, PC9, H460, H82, H1650, H1299, 16HBE	Meng et al. (2022)
circ-IARS	Up	miR-1252-5p	*In-vivo, in-vitro*	H1299, A549, H460, HEK-293T	Yang et al. (2023d)
Circ_0110498	Up	miR-1287-5p	*Human, in-vivo, in-vitro*	A549 and H1299, 16HBE	Hao et al. (2023)
Circ_0043256	Down	miR-1206	*Human, in-vivo, in-vitro*	PC9, A549, Beas‐2B	Zhou et al. (2023)
circ_0000520	Up	miR-1258	*Human, in-vivo, in-vitro*	NCI-H1299, A549, H460, NCI-H2106, H1975, BEAS-2B	Han et al. (2022)
Circ-EIF3I	Up	miR-1253	*Human, in-vivo, in-vitro*	H1650, H460, A549 and H1299, HBE	Chen et al. (2022a)
circ_0003176	Down	miR-182-5p	*Human, in-vivo, in-vitro*	16HBE, H1299, H446, 95-D, A549	Yang et al. (2022a)
Circ_0000808	Up	miR-1827	*Human, in-vivo, in-vitro*	HCC827, A549, NCI-H1299, BEAS-2B, PC9	Cai et al. (2022a)
circ_0018189	Up	miR-656-3p	*Human, in-vivo, in-vitro*	BEAS2B, HCC44, A549	Cai et al. (2022b)
Circ_0002476	Up	miR-1182	*Human, in-vivo, in-vitro*	H1299, A549, 16HBE	Wang et al. (2022b)
circ_0009043	Up	miR-148a-3p	*Human, in-vivo, in-vitro*	A549, HCC827	She et al. (2022)
circFARSA	Up	miR-15a-5p	*Human, in-vivo, in-vitro*	BEAS-2B, HCC827, NCI-H1299, NCI-H23, H125293 T	Nie et al. (2022)
Circ_0001955	Up	miR-769-5p	*In-vitro, human*	BEAS-2B, H520, A549, H358, H460, HCC827	Ding et al. (2022)
circ_0000317	Down	miR-494-3p	*In-vitro, human*	BEAS-2B, A549, H460, PC9, H1299, SPC-A1, HEK293T	Xia and Zhang (2022)
circ_0079530	Up	AQP4	*Human, in-vivo, in-vitro*	H2170, A549, MRC-5	Yang et al. (2022b)
circ_0000518	Up	miR-330-3p	*Human, in-vivo, in-vitro*	A549, H1299, 293T	Lv et al. (2022)
Circ_0010235	Up	miR-588	*Human, in-vitro*	H1650, A549, H1299, HBE	Zhu et al. (2022b)
EPB41	Up	miR-486-3p	*Human, in-vivo, in-vitro*	BEAS-2B, PC9, A549, H1975, H1650	Jin et al. (2022)
circ_0006427	Down	miR-346	*Human, in-vitro*	HNBEC, BEAS-2B, L9981, A549, H292, NCI-H460, H460	Sun et al. (2022)
Circ_0092012	Up	miR-635	*Human, in-vivo, in-vitro*	H460, H1299, A549, HBE, 293 T	Yan et al. (2022)
circ_0006692	Up	miR-205-5p	*Human, in-vitro*	A549, H1299, BEAS-2B, HCC827, H358, PC-9	Liao et al. (2022)

## Exosomal non-coding RNAs and lung cancer

Various research has emphasized the significance of exosomes in both the wellbeing and ailment of humans. Furthermore, more recent investigations have showcased the ability of exosomes to perform their functions through impacting immune response, oxidative stress, autophagy, gut microbiota, and cell cycle (Li C. et al., 2021). Recent research has shown that exosomal ncRNAs are closely linked to the advancement of various human cancers, such as lung cancer. These particular ncRNAs present in exosomes contribute to crucial processes in cancer development, such as EMT, cell growth, formation of new blood vessels (angiogenesis), spread of cancer to other parts of the body (metastasis), resistance to treatment, and immune-inflammatory responses (Hu et al., 2020).

In the field of cell biology, three categories of extracellular vesicles are distinguished by their size and the process involved in their liberation: apoptotic bodies (larger than 1,000 nm), microvesicles (measuring between 100 nm and 1,000 nm), and exosomes (whose length varies between 30 nm and 150 nm) (Shen et al., 2023). Exosomes are small vesicles that resemble a saucer in shape, having two lipid layers. They play a vital role in communication and maintaining balance between cells by transporting their contents from 1 cell to another (Ruzycka-Ayoush et al., 2023). Cell surfaces are specifically targeted by adhesion molecules and tetraspanin complexes (Rana et al., 2012). The SNAREs complex and ESCRT machinery promote the merger of cellular components, using four diverse proteins (sequentially called ESCRT type 0-III) to produce exosomes in a crucial capacity (Henne et al., 2013).

Exosomes can be identified in multiple types of bodily fluids such as blood, urine, breast milk, and bronchoalveolar lavage fluid (Ruzycka-Ayoush et al., 2023). The results of the cell vesicular trafficking process, typically beginning with the fusion of two membranous objects in late endosomes, are products of this machinery (Hessvik and Llorente, 2018). The biogenesis of exosomes progresses through three stages: initially the plasma membrane inward folds creating an early endosome; subsequently, the endosome membrane bulges out forming multiple vesicular bodies (MVBs); and finally, the late endosome fuses with the plasma membrane and expelling MVBs (Batista et al., 2011).

The production of exosomes is a carefully controlled activity which is impacted by the cell type, the environment, and other cells. For instance, mesenchymal stem cells will produce substantially more exosomes than immature dendritic cells. Furthermore, when surrounded by a stressful environment like hypoxia, the release of exosomes increases. It is also known that cells which have contact inhibition and those that are resting will have much lesser amounts of exosomes released (Hayes et al., 2005; Gurunathan et al., 2019). It has been demonstrated that cells with higher division rates, such as cancerous cells, produce and release more exosomes (Liu S.-L. et al., 2019).

Exosomes, which are enclosed in membranes, are found in a variety of biological liquids. These vesicles serve as key communicators between cells and controllers of biological processes. Their loads are complex, consisting of proteins, lipids, DNA, mRNA, and miRNA (Jabalee et al., 2018). One of the most interesting and widely examined molecules are miRNAs, which are short, internal, non-coding RNAs. Their role in regulating gene expression after transcription and translation is remarkable and has attracted considerable attention (Zhang J. et al., 2015; Jabalee et al., 2018) (Figure 4). Evidence suggests that miRNA exchange among tumor and stromal cells within the tumor microenvironment, especially with regards to lung cancer, could be responsible for the initiation and development of the cancer (Adi Harel et al., 2015; Liu Y. et al., 2016; Sun et al., 2018; Xu et al., 2018; Zhang et al., 2019a).

Angiogenesis, which is essential for the expansion and spread of tumors, is regulated by exosomes that are released by different cell types and act as messengers between cells (Zhang L. et al., 2015). A recent study suggested that exosomal miR-9 may encourage blood vessel growth through the activation of the JAK/STAT signaling pathway (Zhuang et al., 2012). The tissue inhibitor of metalloproteinases-1 (TIMP-1) increased exosomal miR-210 originating from lung adenocarcinoma (LUAD) samples, leading to the promotion of angiogenesis in stromal cells (Cui et al., 2015). Liu et al. also found that miR-21, which is located within exosomes, activated signal transducer and activator of transcription (STAT) 3, which then led to enhanced expression of vascular endothelial growth factor (VEGF) and the transformation of normal human bronchial epithelial cells (HBECs) into malignant cells (Liu Y. et al., 2016). At the end, it was shown that exosomal miR‐23a obtained from lung cancer cells had the capability of boosting tumour vascularization in environments with either normal or low oxygen, suggesting that lung cancer cells are capable of passing on genetic material to remote endothelial cells (Hsu et al., 2017).

Immune checkpoint proteins are extremely important in controlling the immune system in order to keep the body in balance and keep it from attacking itself (Smolle et al., 2019). Treating advanced NSCLC patients with medications which focus on the immune checkpoint molecules PD-1 and PD-L1 has been linked to greater lifespans (Arbour and Riely, 2019). Research has revealed that miRNAs can act as a network to regulate processes associated with immune checkpoint pathways. One example is miR-34, which is managed by p53 and directly binds to the PD-L1 3′UTR, ultimately suppressing its presence in non-small cell lung cancer models (Cortez et al., 2016). The function of MiR‐200 was additionally found to regulate the expression of PD‐L1 (Chen et al., 2014).

More and more research suggests that exosomes play a role in cancer growth by transferring molecules and substances that weaken the body’s defences (Greening et al., 2015). MiRNAs that are packaged into exosomes are pivotal in modulating the activities of various immune cells, particularly dendritic cells and T-lymphocytes, in the context of cancer (Sun et al., 2018). For instance, as mentioned above, exosomes from lung cancer cells have been seen to transfer miR‐21/29a to set off TLR7 and TLR8 on immune cells, which could be linked to cancer proliferation and metastasis (Fabbri et al., 2012). Yang et al. found that miR-214 was transferred from human cancer cells, including those related to lung cancer, to recipient CD4^+^ T cells, mediated by exosomes. The result of this was a decrease in PTEN expression and an increased rate of Treg expansion, as well as an increased tumour growth (Yin et al., 2014).

PTEN has an anti-tumor effect on multiple types of cancer, achieved through its ability to inhibit the PI3K/Akt signaling pathway (Tang et al., 2020; Li et al., 2021b). Conversely, EZH2 is capable of attaching to the promoter region of genes like PTEN to alter their expression levels (Pappas et al., 2021). It has been observed that the exosomal long non-coding ribonucleic acid UFC1 presents a hindrance to apoptosis and the termination of the cell cycle in lung cancer cells, and its presence is correlated with a marked rise in cellular proliferation and metastasis. This effect is thought to take place through UFC1’s interference with enhancer of zeste homolog 2 (EZH2), which consequently causes PTEN expression to drastically diminish, thus prompting the forming of conditions necessary for the manifestation of non-small-cell lung cancer (Zang et al., 2020). In addition, lncRNAs that are carried in exosomes can cause lung cancer to become resistant to drugs. Specifically, the lncRNA RP11–838N2.4 can be found in exosomes, thus reducing the effectiveness of erlotinib chemotherapy. Interestingly, the FOXO1 protein has been observed to attach to the promoter of RP11–838N2.4, thereby recruiting histone deacetylases that lessen the expression of RP11–838N2.4 and consequently raise the sensitivity of the cancer to erlotinib chemotherapy (Zhang W. et al., 2018). Comparatively, the exosomal lncRNA GAS5 has been established to act as a tumor-suppressor, but its amounts are substantially lower in individuals with NSCLC. Regrettably, the overexpression of GAS5 can cause lymph node metastasis in advanced stages, resulting in a grim prognosis (Li et al., 2019). In conclusion, DLX6-AS1 and SNHG15, two exosomal lncRNAs with tumor-promoting properties, are found to be expressed at higher levels in lung cancer patients, resulting in a worse prognosis (Table 4) (Zhang et al., 2019b; Stamm et al., 2021).

**TABLE 4:** Various exosomal ncRNAs in lung cancer.

Cargo	Target	Method	Cell line	Ref
miR-21	IRF1	*Human, in-vivo, in-vitro*	H1299, THP-1	Jin and Yu (2022)
miR-155, miR-196a-5p	RASSF4	*Human, in-vivo, in-vitro*	A549, THP-1	Li et al. (2021c)
miR-3157-3p	TIMP	*Human, in-vivo, in-vitro*	H1299, SPCA1, PC9, A549, 16HBE	Ma et al. (2021)
miR-20a	PTEN	*Human, in-vivo, in-vitro*	A549, H838, HCC827, H1299	Shi et al. (2022)
miR-563	-	*Human, in-vivo, in-vitro*	A549	Gao et al. (2022)
miR-197-3p	TIMP2/3	*Human, in-vivo, in-vitro*	SK-LU-1, H358, H1650, H2030, H1975, A549, HCC827, PC9, H2009, H1299	Chang et al. (2022)
miR-338-3p	CHL1	*Human, in-vitro*	BEAS-2B, A549, SK-MES-1	Tian et al. (2021)
miR-7-5p	MNK	*Human, in-vivo, in-vitro*	A549	Liu et al. (2022c)
miR-31-5p	SATB2-reversed EMT	*Human, in-vivo, in-vitro*	A549, H1299, H292, H1975	Yu et al. (2021)
miR-3180-3p	FOXP4	*In-vivo, in-vitro*	A549	Chen et al. (2021a)
miR-1246	DR5	*In-vitro*	A549, SK-MES-1, NCI-H446	Yuan et al. (2016)
miR-375-3p	claudin-1	*Human, in-vivo, in-vitro*	HTB-171, CRL-5853, HEK293T	Mao et al. (2021a)
miR-224-5p	androgen receptor (AR)	*Human, in-vivo, in-vitro*	95-D, PC-9, H1975, H1299, A549, BEAS-2B, HEK293T	Zhou et al. (2021b)
miR-27b	EGFR	*Human*	-	Cao et al. (2022)
miR-133a-3p	SIRT1	*Human, in-vivo, in-vitro*	A549	Yang et al. (2023e)
miR-210	PTEN	*In-vitro*	H1975, A549, BEAS-2B	Yang et al. (2020b)
miR-660-5p	KLF9	*Human, in-vivo, in-vitro*	H1299, H460, A549, H358, 16HBE	Qi et al. (2019)
circSATB2	miR-330-5p	*In-vivo, in-vitro*	H1299, H460, A549	Zhu et al. (2022c)
circSHKBP1	miR-1294	*Human, in-vivo, in-vitro*	HBE, A549, PC9, H1650, H1299	Chen et al. (2022b)
circDNER	miR-139-5p	*Human, in-vivo, in-vitro*	BEAS‐2B, HCC827, A549, H1975, H1299, H460	Li et al. (2022a)
circSATB2	miR-326	*Human, in-vitro*	BEAS-2B, A549, H460, H1299, H226, MES-1, HEK-293 T	Zhang et al. (2020c)
circUSP7	miR-934	*Human, in-vivo, in-vitro*	NCI-H460, NCI-H1299, A549, PC9, 95D	Chen et al. (2021b)
circVMP1	miR-524-5p	*Human, in-vivo, in-vitro*	A549, H1299, 293 T	Xie et al. (2022)
Circ-MEMO1	miR-101-3p	*Human, in-vivo, in-vitro*	H1650, PC9, H1299, A549, HBE	Ding et al. (2020)
circ_0014235	miR-520a-5p	*Human, in-vivo, in-vitro*	A549, H1299, 16HBE	Xu et al. (2020)
circ-IARS	miR-1252-5p	*Human, in-vivo, in-vitro*	BEAS-2B, H1299, A549, H460, (HEK) 293T	Yang et al. (2023d)
circ_0008717	miR-1287-5p	*Human, in-vivo, in-vitro*	A549, H1299, BEAS-2B	Wang et al. (2022c)
circ_0000519	miR-1258	*Human, in-vivo, in-vitro*	H2170, H1299, A549	Wang et al. (2022d)
circERBB2IP	miR-5195-3p	*Human, in-vivo, in-vitro*	A549, H1299, BEAS‐2B	Peng et al. (2023)
lnc-MMP2-2	MMP2	*In-vitro*	A549	Wu et al. (2018)
HOTAIRM1	SPON2	*Human, in-vitro*	A549, H1299	Chen et al. (2022c)
AGAP2-AS1	miR-296	*Human, in-vivo, in-vitro*	THP-1, BEAS-2B, A549, H157, LTEP-2, NIH-H358, SPCA	Zhang et al. (2021c)
UFC1	EZH2	*Human, in-vivo, in-vitro*	A549, H1299, MRC-5	Zang et al. (2020)
LINC00963	Zeb1	*Human, in-vivo, in-vitro*	BEAS-2B, H1975, NCI-H1299, A549, Calu-3, NCI-H358, NCI-H1650	Hu et al. (2023b)
RP5-977B1	-	*Human*	-	Min et al. (2022)
FOXD3-AS1	ELAVL1	*In-vitro*	A549	Mao et al. (2021b)
lnc-MMP2-2	miR-1207-5p	*In-vivo, in-vitro*	A549	Wu et al. (2021)
SCIRT	miR-665	*Human, in-vivo, in-vitro*	BEAS-2B, HBE, H1975, H1650, H446	Wang et al. (2021)
HAGLR	-	*Human*	-	Rao et al. (2019)
MALAT1	miR-613	*Human, in-vivo, in-vitro*	A549, H1299, BEAS-2B	Wang et al. (2020c)
FGD5-AS1	miR-944	*Human, in-vitro*	H358, H1299, PC-9, A549, BEAS-2B	Lv et al. (2021)

**FIGURE 5:**
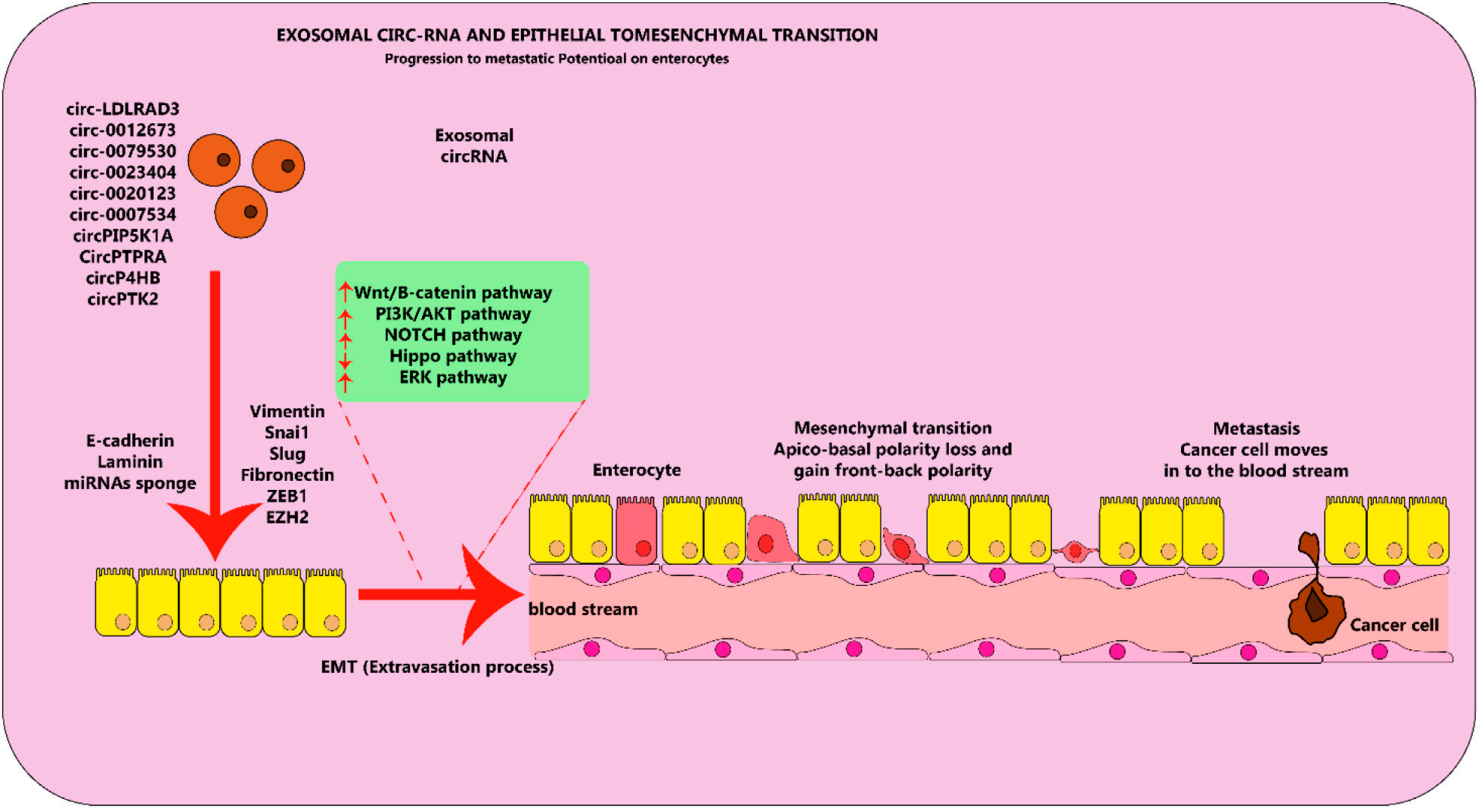
Exosomal circRNA molecules play an important role in the EMT process in lung cancer cells. Circular RNA molecules found in exosomes, which are elevated in levels and contribute to cancer-causing actions, facilitate the process of EMT by stimulating or hindering various pathways in lung cancer. This figure adapted from (Hussen et al., 2022).

Exosomal circRNAs are pivotal in multiple biological functions which can either stimulate or restrict cancer development (Huang X. Y. et al., 2020; Yang et al., 2021). Evidence is mounting that exo-circRNAs have an essential part to play in a range of malignancies, including lung cancer, through various pathways. It has been proposed that exosomal circRNAs function similarly in cancerous cells, where they act like miRNA sponges (Wang et al., 2020b). When epithelial cells become able to move, the process known as EMT occurs and they transform to a mesenchymal state while still having the capability to be invasive (Kalluri and Weinberg, 2009). Many biological occurrences, like the development of an embryo, the forming of scar tissue, the spread of cancer, and metastasis, have been studied by using this approach (Prieto-García et al., 2017). The EMT process can cause LC, a type of malignant tumor, to spread and invade surrounding tissue just as other malignant tumors do (Bakir et al., 2020). A substantial concentration of circRNAs is evident in lung cancer, and certain of them demonstrate cancer-causing roles by spurring EMT activities *in vitro* (Figure 5). Take for instance, the suppression of microRNA-137 by circ-LDLRAD3 augmented the proliferation and EMT through the elevation of glutamine transporter, a portion of the SLC1A5 family, in lung cancer cells (Xue et al., 2020). It was discovered that deactivating SLC1A5, an essential element in the formation and maintenance of LC cells, significantly reduced their survivability (Hassanein et al., 2013). The circulatory function of circ 0012673 may be responsible for the spread and entry of LUADs (Qin et al., 2020). Decreasing the amounts of circ 0012673 interfered with cell development, motility, and EMT by boosting LIM domain kinase 1 in lung adenocarcinoma cell lines, which also caused apoptosis mediated by miR-320a in targeting (Qin et al., 2020). Li et al. discovered that elevating the amount of hsa circ 0079530 can stimulate cancer cells to permeate and proliferate through controlling EMT activities (Li J. et al., 2018).

## Exosomal piRNAs and lung cancer

Piwi-interacting RNAs (piRNAs) are a newly identified type of small non-coding RNAs (sncRNAs), ranging from 24 to 31 nucleotides (nt) in length, that have a crucial role in cancer diagnosis by affecting gene expression. These molecules bind to the PIWI protein family and act as gene regulators, controlling transcriptional activity, preserving the function of germline and stem cells, and regulating translation and mRNA stability (Li et al., 2021d).

Li and colleagues indicated the abnormally manifested piRNAs in lung adenocarcinoma (LUAD), and suggested that the serum exosomal piR-hsa-26925 and piR-hsa-5444 may have promising utility as diagnostic biomarkers for LUAD (Li et al., 2021d). In their analysis, it was determined that 76 piRNAs displayed elevated expression while 9 piRNAs showed reduced expression in LUAD tissues compared to non-tumor tissues. The 10 most noteworthy overexpressed piRNAs were selected and upon conducting qRT-PCR, it was confirmed that 4 of them (piR-hsa-26925, piR-hsa-5444, piR-hsa-30636, and piR-hsa-8757) were significantly upregulated in LUAD tissues. Furthermore, the levels of piR-hsa-26925 and piR-hsa-5444 were significantly higher in the serum exosome samples of LUAD patients compared to those of healthy controls. To conclude, they developed a 2-piRNA panel composed of piR-hsa-26925 and piR-hsa-5444, which displayed superior diagnostic accuracy for LUAD with an AUC value of 0.833 (Li et al., 2021d).

In order to successfully detect NSCLC at an early stage, Li and colleagues conducted a thorough investigation of serum-based extracellular vesicle piRNA in order to identify potential diagnostic indicators for the disease (Li Y. et al., 2022). Researchers used a high-throughput sequencing method to identify potential piRNA biomarkers in cancerous and noncancerous tissues of patients with NSCLC. These piRNAs were then tested in a sample group of 115 patients (including 95 in stage I) and 47 healthy individuals through a quantitative real-time PCR. They found that piR-hsa-164586 was significantly increased compared to noncancerous tissues and extracellular vesicles from healthy individuals’ serum samples. Its diagnostic value was also validated, with an area under the curve (AUC) of 0.623 and 0.624 for distinguishing all stages of NSCLC and stage I specifically from healthy individuals. Compared to a commonly used biomarker, CYFRA21-1, piR-hsa-164586 showed better diagnostic performance. The piR-hsa-164586 was also correlated with clinical characteristics of NSCLC patients, such as age and TNM stage, making it a promising biomarker for early detection of NSCLC (Li Y. et al., 2022).

## Exosomal snoRNAs and lung cancer

SnoRNAs, which are RNA molecules that do not code for proteins, are found in the nucleolus where they play a role in altering and cutting ribosomal RNAs. They were first discovered in the 1960s and extensive research has been conducted to understand their creation and functions. Although their involvement in cancer has only recently been uncovered, studies have revealed that snoRNAs play a significant role in lung cancer. By analyzing the expression of snoRNAs, researchers have been able to define snoRNA-related patterns not only in tissues but also in bodily fluids, suggesting their potential as non-invasive biomarkers. Furthermore, snoRNAs have been found to be crucial in the initiation and spread of lung cancer, influencing a variety of cellular processes such as cell growth and death, and promoting cancer cell adaptability. They possess both cancer-causing and tumor-suppressing qualities that are essential in the development and advancement of lung cancer (Mourksi et al., 2020).

Researching snoRNAs in relation to lung cancer has the potential to lead to novel methods for clinical use, including their use as both biomarkers and targets for treatment. The unique biochemical characteristics of snoRNAs make them an excellent candidate for non-invasive biomarkers in the context of lung cancer. These molecules can be found in exosomes or released from dying cells into bodily fluids such as sputum, making them easily accessible for diagnostic purposes (Su et al., 2016) or plasma (Liao et al., 2010). SnoRNAs can be easily and consistently identified without the need for invasive methods, allowing for their detection in a stable manner. If snoRNA patterns are precisely identified throughout the progression of the illness, this can serve as a significant indicator of both the subtype and the severity of the illness, as well as potentially predicting future relapses (Gong et al., 2017). The identification of new markers will largely depend on the use of RNA-sequencing methods, however, challenges related to the classification of various snoRNA annotations and determining snoRNA identity based on a single 10-20 nt sequence remain to be resolved.

Gao and colleagues (Gao et al., 2015) proposed using RNA-seq to profile small nucleolar RNAs (snoRNAs) in lung cancer. This technology allowed them to analyze 458 mature snoRNAs in 12 pairs of normal and tumor lung tissue from stage I NSCLC patients. They found that 29 snoRNAs, including SNORA71A, were significantly overexpressed (at least 3-fold higher) in tumor tissue compared to normal biopsies. In a separate study, Wang and colleagues investigated the effectiveness of plasma small nucleolar RNAs in diagnosing NSCLC at an early stage. Based on databases, they selected SNORD83A for further analysis and confirmed its expression in both formalin-fixed, paraffin-embedded tissues from 48 paired NSCLC patients, as well as in plasma from 150 NSCLC patients and 150 healthy individuals. Receiver operating characteristic analysis revealed that SNORD83A, alone or in combination with carcinoembryonic antigen, had a high diagnostic efficiency for NSCLC. The levels of SNORD83A were significantly elevated not only in tissues, but also in plasma from NSCLC patients compared to healthy individuals. This indicates that plasma SNORD83A could serve as a diagnostic biomarker for NSCLC. When combined with carcinoembryonic antigen, the diagnostic efficiency for early-stage NSCLC was significantly improved (Wang K. et al., 2022).

## Conclusion and future perspective

To sum up, it has been revealed that lung cancer is an intricate disorder with many unknown regulators. ncRNAs can influence gene expression which in turn can increase or decrease the risk of lung cancer. Examples of ncRNAs that affect these pathways are microRNAs (miRNAs), circRNAs, piRNAs, and lncRNAs. In certain instances, these molecules, such as lnc-JPX, can exercise their influence on multiple pathways. ncRNAs can either be induced or inhibited, which can be utilized to diagnose the early onset of lung cancer, establish the occurrence of metastasis, or foresee recurrent cancer after surgery. Furthermore, certain ncRNAs can either resist or restore sensitivity to medication. For example, miR-21 is known to promote the formation of tumors and occurs in many targets. Nevertheless, the exact mechanisms of action for many of the molecules remain unclear. Some potential future directions for research on ncRNAs in cancer include: 1) Identification and validation of ncRNA biomarkers: Researchers are actively searching for specific ncRNA biomarkers that could help with early detection, diagnosis, and prognostication of cancer. For example, miRNAs have shown promise as potential biomarkers for breast, colorectal, and lung cancer, and other ncRNAs like lncRNAs and circRNAs are also being studied as potential biomarkers. Scientists are currently working on large-scale studies to validate these biomarkers and determine their clinical usefulness. 2) Understanding the role of ncRNAs in cancer progression: Many ncRNAs have been found to play critical roles in cancer progression by promoting tumor growth, invasion, and metastasis. Therefore, further research is needed to fully understand the mechanisms of how these ncRNAs contribute to cancer development and progression, which could lead to the identification of new therapeutic targets. 3) Development of therapeutic strategies targeting ncRNAs: ncRNAs are potential targets for cancer therapy, and there are various methods being explored. One such approach involves using antisense oligonucleotides (ASOs) or small interfering RNAs (siRNAs) to target and degrade specific ncRNAs. Other methods include using small molecules or natural compounds to inhibit the function of specific ncRNAs. Additionally, researchers are exploring the use of CRISPR-Cas9 gene editing to modify ncRNA expression in cancer cells. 4) Combination therapy with ncRNA-targeting agents: Some researchers are investigating the potential of combining ncRNA-targeting agents with other cancer treatments, such as chemotherapy, radiation therapy, or immunotherapy. This combination therapy could potentially overcome resistance to existing cancer treatments and improve patient outcomes.

Recent findings suggest that EVs, specifically exosomal ncRNAs, have been a significant focus of research in lung cancer development in recent years. These EVs contain numerous RNAs that can play a role in oncogenic transfer, angiogenesis, immune modulation, and premetastatic niche formation. Among these EV components, microRNAs and lncRNAs have been shown to dysregulate the expression of molecules involved in epithelial-mesenchymal transition (EMT) and angiogenesis in recipient cells by interacting with signaling pathways. Furthermore, these “harmful” RNAs can also disrupt the microenvironment in secondary organs, leading to increased tumorigenic heterogeneity and the formation of premetastatic niches. Conversely, immune cell-derived exosomal RNAs have a modest impact on the composition and activation of T cells, B cells, and other immune cells, ultimately promoting therapeutic resistance in tumor cells. These EVs also play a crucial role in the maintenance of non-cancer stem cells (CSCs) and CSCs, and further research is needed to fully understand their function in tumors. They have potential as both diagnostic markers and therapeutic targets, allowing for personalized medicine to be developed based on their specific characteristics. However, the challenge of therapeutic resistance during cancer treatment remains a hurdle. Additionally, more research is needed to fully understand the effects and regulation of EV-derived RNAs, as well as ensure the safety and quality of new methods for their isolation and use. As we continue to gain a better understanding of EVs, more emphasis will likely be placed on *in vivo* models and clinical applications to further address these questions.
